# Cognitive synergy in groups and group-to-individual transfer of decision-making competencies

**DOI:** 10.3389/fpsyg.2015.01375

**Published:** 2015-09-22

**Authors:** Petru L. Curşeu, Nicoleta Meslec, Helen Pluut, Gerardus J. M. Lucas

**Affiliations:** ^1^Department of Psychology, “Babeş-Bolyai” UniversityCluj-Napoca, Romania; ^2^Department of Organisation Studies, Tilburg UniversityTilburg, Netherlands; ^3^Strategy, Beedie School of Business, Simon Fraser UniversityBurnaby, BC, Canada

**Keywords:** decision-making competencies, heuristics and biases, cognitive synergy, group rationality

## Abstract

In a field study (148 participants organized in 38 groups) we tested the effect of group synergy and one's position in relation to the collaborative zone of proximal development (CZPD) on the change of individual decision-making competencies. We used two parallel sets of decision tasks reported in previous research to test rationality and we evaluated individual decision-making competencies in the pre-group and post-group conditions as well as group rationality (as an emergent group level phenomenon). We used multilevel modeling to analyze the data and the results showed that members of synergetic groups had a higher cognitive gain as compared to members of non-synergetic groups, while highly rational members (members above the CZPD) had lower cognitive gains compared to less rational group members (members situated below the CZPD). These insights extend the literature on group-to-individual transfer of learning and have important practical implications as they show that group dynamics influence the development of individual decision-making competencies.

## Introduction

*“…a group is an aggregate of organisms in which the existence of all is utilized for the satisfaction of some needs of each” (Cattell et al., 1953, p. 332)*.

Modern organizations often appoint groups to make decisions with important organizational or societal consequences and in spite of the increasing scholarly interest in decision-making competencies we know relatively little about how group interactions change individual decision-making competencies. Decision competencies reflect the (individual or group) capability of consistently making choices aligned with a normative ideal (Parker and Fischhoff, 2005; Bruine de Bruin et al., 2007; Baron, 2012; Curşeu and Schruijer, 2012a), a notion inspired from the classic view on rationality (Shafir and LeBoeuf, 2002). As attempts to bolster individual decision competencies by teaching about game theory and econometrics were unsuccessful (Rubinstein, 1999), an important question remains on how to train individual decision competencies. In this paper, we aim to examine whether group-to-individual transfer of learning generated via specific competence-based group configurations and synergetic interpersonal processes in groups can improve individual decision-making competencies.

The current study builds on the group-to-individual learning transfer literature (Laughlin and Sweeney, 1977; Olivera and Straus, 2004; Laughlin et al., 2008; Schultze et al., 2012) to argue that social and cognitive factors influence group-to-individual learning transfer and in particular the development of individual decision-making competencies. Groups are multi-level social systems with cognitive and emotional emergent properties (Barsade and Gibson, 1998; Curşeu, 2006; Woolley et al., 2010; Hinsz, 2015). By interacting with each other in groups, individual group members change their cognitive structures and competencies and generate collective, group level cognitive structures and competencies (Curşeu et al., 2007). This cognitive co-evolution process (also labeled collective induction) is argued to be the source of group cognition (Curşeu, 2006; Curşeu et al., 2007; Hinsz, 2015) as well as of individual cognitive change and development (Goos et al., 2002).

In line with the multi-level theory of groups (Klein and Kozlowski, 2000; Kozlowski and Chao, 2012; Kozlowski et al., 2013), two top-down group level factors are expected to influence individual cognitions. We distinguish between compositional and compilational factors because individuals may learn from other group members (compositional effects) as well as from the group as a whole (compilational effects) (Brodbeck and Greitemeyer, 2000). First, compositional systemic properties reflect the within group variability (or constellation) of individual (cognitive) competencies and the focus lies on an individual's position in relation to other group members. In compositional terms, the development of individual decision competencies varies as a function of the individual's distance from the other group members. Second, compilational systemic properties describe the system as a whole and transcend individual (cognitive) competencies. Group level emergent decision competencies may shape the competencies of the individual group members. In order to capture such compilational effects, we focus on group synergy and argue that the development of individual decision competencies depends on the effectiveness of the collective induction processes (that is, the extent to which groups generate cognitive phenomena that transcend the individual cognitions of their members).

## Group-to-individual (G-I) transfer of learning

G-I transfer of learning describes the extent to which individual group members improve their specific knowledge, problem solving, or decision-making competencies after engaging in collaborative tasks (Laughlin and Sweeney, 1977). In the G-I transfer literature, researchers typically compare subsequent individual performance for participants who first performed tasks (decision-making or problem solving) individually versus in teams. For example, Schultze et al. (2012) performed two studies in which they compared the performance of interacting groups with the average (prior to group task) performance of their individual members in quantitative judgment tasks. Their results showed that groups' superiority to individual performance was solely attributable to individual cognitive gains following group interactions.

As idea generation is essential in decision making (DelMissier et al., 2015), we argue that interpersonal interactions in groups create collaborative zones of proximal development that facilitate the development of individual decision competencies. We posit that through social learning processes less competent group members can develop their decision competencies by interacting with more rational group members. The position a member has in the group in terms of rationality shapes the learning potential of that group member (compositional effect). In addition, effective collective induction processes generate group level decision-making competencies that transcend individual competencies, and individual group members benefit from these emergent group level competencies (compilational effect). We further elaborate on these two mechanisms for G-I transfer of learning in subsequent sections.

### Social learning and collaborative zones of proximal development

We draw on the concept of “zones of proximal development” to explain social learning processes in groups. The notion of zones of proximal development illustrates the distance between the actual developmental level of a person and the level of potential development as determined by collaboration with more capable peers (Vygotsky, 1978). Goos et al. (2002) extended the concept of zone of proximal development and argued that small groups develop *collaborative* zones of proximal development (CZPD). As such, the configuration of individual decision competencies within a group creates potential CZPD. These potential CZPD emerge around the average of individual competencies, as Goos et al. (2002) argued that “there is learning potential in peer groups where students have incomplete but relatively equal expertise—each partner possessing some knowledge and skill but requiring the others' contribution in order to make progress” (p. 195). Collaborative zones of proximal development are therefore necessary in order for the small group to be effective in helping individual members develop their decision-making competencies.

In the compositional perspective, individual group members learn from each other through vicarious observation and peer learning effects. However, the effectiveness of social learning processes among group members varies as a function of the similarity of the knowledge repertoires held by those involved in the learning process. In other words, individual group members benefit differently from social learning processes depending on their relative position in relation to the potential CZPD and, in line with the complementary-task type model (Steiner, 1966), group members would benefit especially if they work with partners of similar or higher competence (Laughlin et al., 2008).

Small groups stimulate meta-cognitive activities and strategies as individual group members engage in social comparisons and develop their own insights into the cognitive processes underlying the evaluation of alternatives in decision-making (McNeese, 2000; Hinsz, 2015). These socially induced meta-cognitive activities may lead to the improvement of individual decision competencies following interpersonal interactions in groups. Group members situated below the CZPD can benefit from the CZPD created by other more competent members. In line with Vygotski's cognitive development theory, the collaborative zones of proximal development are situated above the competence of the least rational group member and represent a zone of potential development for this individual (Chaiklin, 2003). Through social learning processes the least rational group members will manage to improve their rationality from an initial level to the level of their potential. Through vicarious observation less competent members copy the decision-making strategies displayed by more competent members (Manz and Sims, 1981) and through peer learning less competent members improve their knowledge repertoires and cognitive skills by interacting with more competent group members (Steiner, 1966; Davis and Luthans, 1980).

In the proximal condition, the differences in rationality among the group members are rather low and individuals are situated around the CZPD. That is, group members are situated relatively close to each other and for this reason they create an accessible source of development. Nevertheless, given that rationality differences are not that high, we expect that the potential for social learning is lower as compared to individuals situated below the CZPD. Group members situated above the CZPD are less likely to benefit from reciprocally exploring each other's reasoning and decision-making strategies (Steiner, 1966). In consensus-based decision-making, group members situated above the CZPD could even experience a deterioration of their decision-competencies. If group members have to agree on joint evaluations, rational members may be misled by the insights shared by the less rational members. On the basis of these theoretical arguments, we expect that the cognitive benefits of collective decision-making efforts for individual group members (i.e., G-I transfer of learning) are dependent on an individual's position in relation to the collaborative zones of proximal development. More specifically, we argue that learning potential varies as a function of ones' position in relation to the CZPD.

*Hypothesis 1: One's relative position in relation to the CZPD influences the development of decision competencies in such a way that group members with lower rationality than the rest of the group members have a higher cognitive gain compared to group members having a higher rationality than the rest of the group*.

### Collective induction and group synergy

Collective induction is a group cognitive process by which members' individual responses or preferences are combined and recombined through social interaction in such a way that the group as a whole generates solutions and alternatives that could not have been generated through a simple aggregation of individual responses. In other words, collective induction is a form of generative learning (McNeese, 2000) through which new knowledge structures (solutions, decisions, judgments) emerge from the cognitive combinatorial process that unfolds during social interactions. The results of collective induction are collaborative in nature and exceed what individual group members could learn, generate, understand, or infer alone (Laughlin and Barth, 1981). The outcome of collective induction is what has been coined in the literature as group synergy.

The group synergy framework has been used to describe the emergence of group rationality as a collective decision-making competence (Curşeu et al., 2013, 2014; Meslec and Curşeu, 2013). Groups that achieve weak synergy are those groups that collectively are more rational than the average rationality of their individual members, while groups that achieve strong synergy are those groups that collectively are more rational than their most rational group member. The concept of group synergy therefore captures the effectiveness of the collective induction processes, in that groups that exceed their average or their best member are those in which the generative learning was most effective.

In a compilational perspective, the group as a whole develops—through collective induction—new knowledge structures or competencies that transcend those of their individual members. Through synergy, groups create new collaborative zones of proximal development, thus creating new learning opportunities for all group members. Groups that achieve weak synergy manage to reach levels of performance that are higher than the average performance of the group members, which translates into a new CZPD that moves above the average performance of the group members and creates higher learning potential for the average group members but still not for the best member in the group. When groups achieve strong synergy, the CZPD moves above the level of the best member in the group and therefore creates a learning opportunity for all group members, even for the most rational individual. This notion is consistent with previous research indicating that individuals who were part of a successful group performed significantly better in a subsequent similar task than individuals who were part of an unsuccessful group (Barron, 2003). Given the reasoning above, we put forward the following hypothesis.

*Hypothesis 2: Group synergy fosters the development of individual decision-making competencies in such a way that the strength of synergetic processes in groups (reflecting the effectiveness of collective induction) has a positive influence on the individual cognitive gain*.

## Methods

### Participants

We sampled 148 students (74 women and 74 men, *M*_age_ = 19.1 years) enrolled in an undergraduate course at a Dutch university (144 participants had no missing data and were included in the final analyses). Participants were informed that they would take part in a collaborative learning exercise aimed at illustrating the relationship between individual and collective decision-making competencies. The collaborative learning exercise was part of regular curricular activities in a workshop devoted to decision-making. We first asked participants to perform a set of decision tasks individually and then they were asked to do the same decision tasks in small groups. These groups (38 in total with an average size of 3.89 members) were previously formed and had a stable membership throughout the semester, thus our study uses actual groups composed of members that previously interacted in the past and had a foreseeable future together. Verbal consent was asked before the start of the workshop, and participants were informed that their results would be used in scientific research and that they could request their data being excluded from said research. Because this exercise was part of curricular activities, no foreseeable risks, beyond those present in regular curricular activities in higher education, were anticipated in this study. However, participants were informed that they could contact the lecturer if they experienced problems with the exercise. According to the Dutch national ethical guidelines, studies based on questionnaires that do not require any personal data with the potential to embarrass the participants, and educational studies aimed at exploring knowledge or skill acquisition in educational settings are exempted from ethical committee approval, therefore no supplementary approval was asked from the local IRB.

### Procedure

At the beginning of the workshop, participants were asked to individually fill out 10 decision tasks, on the basis of which we evaluated individual rationality at time 1 (IRT1). Then they were asked to redo the same decision tasks collectively in small groups. Based on the collective decisions we evaluated group rationality (GR). Finally, after the group decision-making, the individual group members were asked to fill out a set of 10 decision tasks that stemmed from the same task domain but were different from those used in T1. Based on these tasks we evaluated individual rationality at time 2 (IRT2). This procedure fits group synergy research (Larson, 2007; Curşeu et al., 2013, 2014; Meslec and Curşeu, 2013) as it allows for computing weak and strong group synergy. It also aligns well with the procedures used in group-to-individual transfer of learning research (Schultze et al., 2012). After completing the second set of decision tasks, we explained the aim of the exercise to the participants. During the debriefing, we explained all heuristics and biases used in the decision tasks. We asked participants to compute their individual and group scores and we guided them in reflecting on how individual decision competencies are related to the emergent group decision competencies.

### Instruments

***Decision-making competencies*** at time 1 were evaluated with a set of 10 decision tasks previously used to evaluate decision rationality (Curşeu and Schruijer, 2012a). The 10 decision-making tasks were adapted from experimental procedures used to illustrate various biases in the decision-making literature, namely the framing effect (2 items), representativeness bias (6 items), and Ellsberg's paradox (2 items). The items were formulated as multiple choice items and the normatively correct alternative was always presented among the alternatives participants had to choose from. Individual and group decision competencies were computed by summing the number of normatively correct answers (selected by individuals and groups, respectively) and the resulting score reflects decision rationality defined as the extent to which individual or group choices are aligned with a normative ideal. Decision competencies at time 2 were evaluated using a set of 10 decision tasks that were different but related to the first 10 decisions. This second set of decision tasks was created in a similar fashion to the tasks used at time 1 and had the same distribution of decision tasks: framing effect (2 items), representativeness bias (6 items), and Ellsberg's paradox (2 items). The items are presented as Supplementary Material (Data Sheet 2). Due to the heterogeneity of items used to evaluate rationality and similar with previously reported data on these decision tasks (Curşeu and Schruijer, 2012a), Cronbach's alpha is rather low (0.43 at time 1 and 0.26 at time 2). The inter-item correlations are presented as Supplementary Material (Data Sheet 1). In general, one can observe higher inter-item correlations within rather than between heuristics. Although reliability is problematic, the construct validity ensured by the fact that the items are derived from experimental procedures used to evaluate deviations from rational behavior supports the use of this approach for assessing decision competence as rationality.

***Cognitive gain*** (CG), one of the dependent variables, was computed as the difference between individual rationality at time 2 and individual rationality at time 1: CG = IRT2-IRT1.

***Group synergy*** was evaluated by comparing the group rationality score with the individual rationality scores at time 1. In line with the procedures specified in Larson (2007) and Bornstein and Yaniv (1998), we made a distinction between members of: (1) non-synergetic groups (groups in which the level of collective rationality was lower than the average level of individual rationality), (2) weak synergy groups (groups in which the collective level of rationality was higher than the average level of rationality of their members but lower than the level of rationality of their most rational member), and (3) strong synergy groups (groups in which the collective level of rationality is higher than the level of rationality of their most rational group member). Based on categorizing participants in these three types of groups, 43 participants (in 13 groups) belong to the non-synergetic group condition, 76 (in 18 groups) to the weak synergy condition and 26 participants (in seven groups) to the strong synergy condition. Because the three conditions reflect an incremental change in collective induction processes we coded with zero participants in non-synergetic groups, with one those in groups with weak synergy and with two the participants in strong synergy groups.

***One's position in relation to the CZPD*** was computed on the basis of the constellation of individual rationality levels in the group. Specifically, we computed the difference between a focal participant's rationality score and the average of the remaining fellow group members' individual rationality scores at time 1. This score does not directly measure the CZPD itself (as it is a continuous variable), but it is a proxy for one's relative position in relation to the average decision competence of the rest of the group.

As ***control variables*** we used age and gender. These variables were found to be related to decision-making competencies and interpersonal interaction style in small groups (Curşeu and Schruijer, 2012a; Curşeu et al., 2013, 2015).

### Randomization checks

In our study, we used established groups in order to better capture synergetic processes and their effect on the development of decision-making competencies (Curşeu and Schruijer, 2012b). Although students were originally randomly assigned to groups, we decided to conduct some randomization checks before running the analyses. We therefore performed an ANOVA with the group ID as factor and the individual rationality at time 1 as dependent variable. We found no significant effect, *F*_(37, 106)_ = 1.49, *p* = 0.06, showing that the between group variance was not significantly different from the within group variance. Moreover, as Levene's test for equality of error variances was not significant, *F*_(37, 106)_ = 1.37, *p* = 0.10, we can conclude based on these joint results that participants were sufficiently randomized across groups in terms of their initial decision competencies.

## Results

Table 1 presents the descriptive statistics and the correlations for the variables included in our analyses; Table 2 presents the descriptive statistics for the synergy subgroups.

**Table 1:** **Means, Standard Deviations, and Correlations**.

	**Mean**	***SD***	**1**	**2**	**3**	**4**	**5**	**6**	**7**
1. Gender	0.50	0.50	1						
2. Age	19.10	1.96	−0.21^*^	1					
3. IR T1	3.89	1.71	−0.04	0.06	1				
4. IR T2	4.47	1.57	−0.04	−0.07	0.28^**^	1			
5. GR	4.84	1.51	0.02	0.14	0.28^**^	0.46^**^	1		
6. PCZDP	0.10	1.87	−0.08	0.01	0.80^**^	0.06	0.01	1	
7. GrSYN	0.88	0.68	0.06	0.04	0.07	0.24^**^	0.64^*^	0.004	1
8. CG	0.59	1.96	−0.01	−0.11	−0.64^**^	0.55^**^	0.14	−0.64^**^	0.26^**^

*IRT1, individual rationality at time 1; IRT2, individual rationality at time 2; GR, group rationality; PCZDP, one's position in relation to the CZPD; GrSYN, group synergy coded as 0 = no synergy, 1 = weak synergy, 2 = strong synergy; CG, cognitive gain (computed as IRT2-IRT1); gender is coded with 1 = woman and 0 = man*.

** *p < 0.01*,

* *p < 0.05*.

**Table 2:** **Means and standard deviations for the synergy based subgroups included in the study**.

**Independent variable**	**Level**	**N**	**Cognitive gain**		**IRT2**	
			**Mean**	***SD***	**Mean**	***SD***
Group synergy	No synergy	43	−0.40	1.99	3.82	1.89
	Weak synergy	75	0.55	1.68	4.52	1.61
	Strong synergy	26	2.44	2.97	5.67	2.27

*SD, standard deviation; CZPD, collaborative zone of proximal development; IRT2, individual rationality at time 2; Means and standard deviations are reported based on the ANOVA results with age and gender as controls and for IRT2 also with individual rationality at time 1 as control variable*.

In order to account for the nested nature of the data (individuals nested in groups) we performed multilevel analysis. In addition, to account for gender and age related differences in decision competencies we controlled for gender and age in our analyses. Our data have a multilevel structure, with individuals (Level 1; *n* = 148) nested within groups (Level 2; *n* = 38). We therefore used a hierarchical linear modeling (HLM) framework to perform multilevel analyses (using HLM version 7). HLM takes into account that scores from lower-level units (individuals) are dependent upon membership within higher-level units (groups) and allows for estimating effects of variables at multiple levels of analysis (Bryk and Raudenbush, 1992; Hofmann et al., 2000), thus making it particularly appropriate for our purposes. In addition to entering these control variables, we included relative position to CZPD as a Level-1 predictor and group synergy as a Level-2 predictor to the multilevel equation. One's position in relation to the CZPD was operationalized as a continuous variable and synergy was operationalized using three categories: no-synergy (0), weak synergy (1), and strong synergy (2) groups. The results of the HLM analysis are presented in Table 3.

**Table 3:** **HLM results for cognitive gain**.

	**Cognitive gain**	
	***B* (*SE*)**	***t* (sig)**
**LEVEL-1 PREDICTORS**		
Gender	−0.40 (0.26)	1.53 (0.12)
Age	−0.11 (0.04)	−2.64 (0.01)
One's position in relation to CZPD	−0.68 (0.05)	−13.64 (<0.001)
**LEVEL-2 PREDICTOR**		
Group synergy	0.77 (0.25)	2.96 (0.005)

*Women are coded as 1, men as 0; for synergy 0 = non-synergic groups, 1 = weak synergy, 2 = strong synergy; B = unstandardized HLM coefficient. SE, standard error. A two-level model was tested (individual—group); CZPD, collaborative zone of proximal development*.

The effect of one's position in relation to the CZPD is negative and significant (*B* = −0.68, *p* < 0.001) showing that group members that were more rational than the rest of the group had a lower cognitive gain than group members that were less rational than the rest of the group. Therefore we can conclude that the first hypothesis was fully supported by the data. Moreover, our results showed that group synergy had a significant positive effect on cognitive gain (*B* = 0.77, *p* = 0.005). As the effectiveness of the collective induction processes increases (as illustrated by the strength of the synergetic group processes) so does the cognitive gain experienced by the individual group members. Therefore, the second hypothesis was also fully supported. Additionally, age had a significant negative effect on cognitive gain (*B* = −0.11, *p* = 0.01), while the effect of gender was not significant.

We performed a supplementary multilevel analysis in order to assess the robustness of our findings and to address the issues of endogeneity inherent to our measures. Measures for group synergy, one's position in relation to the CZPD and cognitive gain were all partially derived from individual rationality scores at time 1, implying that our previous results may suffer from an endogeneity bias. We therefore set out to test the impact of group synergy and one's position in relation to the CZPD on individual rationality at time 2. The results of the HLM analysis with individual rationality at time 2 as dependent variable are presented in Table 4.

**Table 4:** **HLM results for individual rationality at Time 2**.

	**Individual rationality T2**	
	***B* (*SE*)**	***t* (sig)**
**LEVEL-1 PREDICTORS**		
Gender	−0.38 (0.25)	−1.52 (0.13)
Age	−0.09 (0.04)	−2.39 (0.01)
Individual rationality T1	0.61 (0.12)	4.88 (<0.001)
One's position in relation to CZPD	−0.40 (0.10)	−3.95 (<0.001)
**LEVEL-2 PREDICTOR**		
Group synergy	0.70 (0.25)	2.78 (0.008)

*Women are coded as 1, men as 0; for synergy 0 = non-synergic groups, 1 = weak synergy, 2 = strong synergy; B = unstandardized HLM coefficient. SE, standard error. A two-level model was tested (individual—group); CZPD, collaborative zone of proximal development*.

The second set of results was consistent with the previous one using the cognitive gain as dependent variable. Individual rationality at time 2 was negatively and significantly predicted by relative position to CZPD (*B* = −0.4, *p* < 0.01) and positively and significantly predicted by group synergy (*B* = 0.7, *p* < 0.01). With respect to the control variables, individual rationality at time 1 positively predicted individual rationality at time 2 (*B* = 0.61, *p* < 0.01), while age had a significant negative effect on individual rationality at time 2 (*B* = −0.09, *p* < 0.05). The influence of gender was not statistically significant (*B* = −0.38, *p* > 0.05). To conclude, the results with individual rationality at time 2 as dependent variable fully replicated the original results using cognitive gain. Therefore we can conclude that our results are robust and both hypotheses are supported by the data.

Finally, following a suggestion made by one of the reviewers, we have performed another robustness check, by using a testing procedure reported in Willis et al. (2010) and predicted individual rationality at time 2 using just individual rationality at time 1 and group rationality as predictors (we controlled for age and gender as well). Although this statistical analysis does not address directly our two hypotheses, it allows us to compare the model fit for the simple model (procedure reported in Willis et al., 2010) and the model we reported in Table 4. In order to obtain the scores for the Akaike's Information Criterion (AIC) and the Schwarz's Bayesian Criterion (BIS) we have run these additional analyses using the mixed models procedure in SPSS. For the model reported in Table 4, AIC = 500.51 and BIC = 506.35. For the simplified model, with individual rationality at time 2 predicted by rationality at time 1 and the group score, we have two positive effects: B = 0.45 (*SE* = 0.09, *t* = 4.63, *p* < 0.001) for group rationality and *B* = 0.11 (*SE* = 0.06, *t* = 1.68, *p* = 0.09) for the influence of individual rationality at time 1 and for this model the AIC = 499.48 and BIC = 505.38. The comparison between the two models reveals rather small differences in the AIC and BIC and the simpler model reveals a strong and significant effect of group rationality on individual rationality evaluated at time 2, supporting our general findings related to the collective induction effects on the development of decision competencies.

## Discussion

Previous research extensively addressed the group-to-individual transfer of learning and documented the superiority of groups as compared to individuals in decision-making tasks (Moshman and Geil, 1998; Schultze et al., 2012; Curşeu et al., 2013; Maciejovsky et al., 2013; Hinsz, 2015).

Thus far, research used collective induction processes to explain such superiority. Surprisingly, no study addressed this claim explicitly hampering scientific progress in this field. In the present study we used two sets of decision tasks adapted from the heuristics and biases literature to assess both individual and collective decision competencies. Building on the group synergy literature (Larson, 2007, 2010; Curşeu et al., 2013; Meslec and Curşeu, 2013), we argued that especially strong cognitive synergy is an accurate indicator of collective induction and we then tested the influence of group synergy on the development of individual decision competencies. Our results show that members of synergetic groups develop their decision competencies through group interaction processes and members of strong synergy groups obtain the highest cognitive benefits.

Next to the alleged collective benefits of cognitive synergy in groups (Larson, 2010), synergetic groups also influence the cognitive skills of their members. Synergy however, and in particular strong cognitive synergy is not easy to achieve Larson, 2007; Curşeu et al., 2013, 2014; Meslec and Curşeu, 2013. Several studies manipulated decision rules in order to test the effect of different patterns of interpersonal interactions on the emergence of strong synergy in groups (Curşeu et al., 2013; Meslec et al., 2014). However, although some decision rules (e.g., a collaborative decision rule) seem to be superior in generating strong cognitive synergy, on average groups did not outperform their worthiest member. Therefore, looking at the empirical evidence to date, strong cognitive synergy is elusive and more research is needed to further identify and explore the factors that could generate strong synergy in groups.

A second important insight of this study refers to the effect of one's relative position in the CZPD on cognitive gains. As hypothesized, our results show that the group members that are situated below or within the CZPD are more prone to learn from the rest of the group. However, the group members that are more rational than the rest of the group could potentially learn only in groups with strong group synergy. This insight points toward the need for generating a novel CZPD through collective induction; in synergetic groups the CZPD is expanded in a way that also benefits the best performing individuals in the group. Future research should explore in a more systematic manner the interplay between the compositional and compilational processes in the transfer of decision-making competencies. More specifically, it is important that future studies find ways to directly induce group synergy and manipulate one's relative position to CZPD in order to further test the effect of the interplay between compilational and compositional processes on the group to individual transfer of decision-making competencies.

On average, individuals that belong to non-synergetic groups experience a cognitive decline, that is, group interaction processes decrease the decision competencies of their members. These results open important venues for further research. In particular it is important to understand the processes through which non-synergetic groups decrease the decision competencies of their most rational members. We advance here a few plausible explanations for these findings. First, dominant members with low decision competencies could have exerted substantial influence and changed the opinions of the other group members (minority domination effect). Second, by discussing inaccurate evaluations of the decision situations, shared by the majority of the group members, the most rational group members could have been misled in their subsequent decisions (majority domination effects). Moreover, the compromise-seeking tendency in group debates that is specific to the Dutch context (Lijphart, 1975) could partially account for the cognitive decline recorded for highly rational participants in non-synergetic groups. Unfortunately, we do not have process data that could have help us test these alternative explanations. Yet, future research could further address these potential explanations. To conclude, our study provides initial empirical evidence concerning the factors that influence the transfer of decision-making competencies from groups to individuals.

### Limitations

Next to its contributions, the present study has some limitations as well. First, we did not record the group discussions, therefore we have no data on the communication and social influence processes unfolding in groups. Second, we have used only a small selection of decision situations from the literature on decision heuristics and biases. Future research could extend the variety of tasks used and attempt to document whether the cognitive gains attributable to cognitive synergy vary across various decision-making tasks and the degree to which these changes are stable in time. Even so, as we selected some of the most common situations in which biases and heuristics are prone to arise, this small set of decision tasks is indicative for the challenges decision-makers (individual and group) face. Third, we have not directly manipulated group synergy nor CZPD (they are observed variables) therefore the causal claims should be treated with caution. Fourth, given the nature of the decision tasks, our results are susceptible to a ceiling effect (individuals with the maximum score at T1 cannot experience a cognitive gain at T2), however, in the whole sample only one participant had the maximum score at T1 and none had the maximum score at T2. Therefore, we can conclude that the ceiling effect is not a likely bias for our results. Fifth, the convenience sampling method and the student population could indicate a selection bias and limit the generalizability of our findings. Future research could attempt to replicate these findings in different samples and address this selection bias. Moreover, specific training in statistics and probability judgment undergone by students could increase the likelihood of success in the 10 decision tasks. However, given that the students in our study were first-year students this seems to be less of a concern than it would have been, had we included later cohorts. Finally, the regression toward the mean could be an alternative explanation for our results concerning the development of the individual decision competencies. In other words, extremely high scores for individual rationality at time 1 could converge toward less extreme scores at time 2. In order to explore this alternative explanation, we have used a repeated measure ANOVA to test the change in the within group SD from time 1 to time 2 using group size as a control (to account for the covariance between group size and within group SD). The results reveal that although the average within group SD is lower at T2 (*M* = 1.05, *SD* = 0.50) as compared to T1 (*M* = 1.42, *SD* = 0.68), the difference is not statistically significant *F*_(1, 36)_ = 0.67 (0.41). This result however cannot fully refute the regression toward the mean alternative explanation.

### Practical implications

Because groups are often required to make important organizational and societal decisions, it is important to understand their impact on the development of individual decision competencies. The use of groups to make important organizational decisions is especially important due to the group superiority effect (in general groups are more rational than the average rationality of their members) (Hinsz, 2015). However, next to the benefits attributable to higher decision quality in groups, our results show that (synergetic) groups are a valuable training ground for individual decision-makers. Our findings show that G-I transfer of learning is influenced by compositional as well as compilational factors. In other words, group members learn from each other as well as from the group as a whole. Special attention should be paid to the quality of interpersonal interactions in groups. Highly rational group members seem to suffer from being part of non-synergetic groups. Moreover, the potential learning they could generate for the group as a whole and for the less rational individuals in particular, is not achieved. Thus, in order to optimize the learning effect, when strong cognitive synergy is not achieved, groups will need tools to identify and improve the performance of their most rational group members. The identification of the most knowledgeable group members (in a particular decision domain) could be facilitated through the use of specific decision rules (Meslec et al., 2014) or the use of decision support systems.

## Funding

PLC was supported by a grant of the Romanian National Authority for Scientific Research, CNCS—UEFISCDI, project number PN-II-ID-PCE-2011-3-0482.

### Conflict of interest statement

The authors declare that the research was conducted in the absence of any commercial or financial relationships that could be construed as a potential conflict of interest.
