# Supplementary material for: Cognitive synergy in groups and group-to-individual transfer of decision-making competencies
Source: Front Psychol. 2015 Sep 22;6:1375. doi: 10.3389/fpsyg.2015.01375 (PMC4585191; doi:10.3389/fpsyg.2015.01375)
Supplement: Supplementary file 1 [file DataSheet1.DOCX]

Inter item correlations for decisions at time 1

|  | 1 | 2 | 3 | 4 | 5 | 6 | 7 | 8 | 9 | 10 |
| --- | --- | --- | --- | --- | --- | --- | --- | --- | --- | --- |
| 1. It 1 T1 | 1,000 | ,085 | ,196 | ,095 | ,192 | ,040 | ,026 | ,022 | -,034 | -,030 |
| 1. It 2 T1 | ,085 | 1,000 | -,061 | -,053 | -,004 | ,155 | ,087 | ,074 | ,351 | -,001 |
| 1. It 3 T1 | ,196 | -,061 | 1,000 | -,016 | ,087 | ,140 | ,107 | ,053 | -,063 | -,011 |
| 1. It 4 T1 | ,095 | -,053 | -,016 | 1,000 | ,082 | ,070 | ,035 | ,326 | ,104 | ,029 |
| 1. It 5 T1 | ,192 | -,004 | ,087 | ,082 | 1,000 | ,161 | ,140 | ,003 | -,016 | ,094 |
| 1. It 6 T1 | ,040 | ,155 | ,140 | ,070 | ,161 | 1,000 | ,214 | -,046 | ,099 | ,085 |
| 1. It 7 T1 | ,026 | ,087 | ,107 | ,035 | ,140 | ,214 | 1,000 | -,087 | ,033 | ,104 |
| 1. It 8 T1 | ,022 | ,074 | ,053 | ,326 | ,003 | -,046 | -,087 | 1,000 | ,216 | ,018 |
| 9. It 9 T1 | -,034 | ,351 | -,063 | ,104 | -,016 | ,099 | ,033 | ,216 | 1,000 | ,098 |
| 10. It 10 T1 | -,030 | -,001 | -,011 | ,029 | ,094 | ,085 | ,104 | ,018 | ,098 | 1,000 |

Note: It 1 T1 = item 1 at time 1, etc

Inter item correlations for decisions at time 2

|  | 1 | 2 | 3 | 4 | 5 | 6 | 7 | 8 | 9 | 10 |
| --- | --- | --- | --- | --- | --- | --- | --- | --- | --- | --- |
| 1.It 1 T2 | 1,000 | ,046 | -,064 | -,001 | ,105 | -,096 | ,092 | ,110 | -,008 | ,042 |
| 1.It 2 T2 | ,046 | 1,000 | -,083 | -,037 | ,046 | -,022 | -,062 | ,035 | ,413 | -,132 |
| 3.It 3 T2 | -,064 | -,083 | 1,000 | -,110 | -,014 | ,045 | ,073 | -,146 | -,105 | ,015 |
| 4.It 4 T2 | -,001 | -,037 | -,110 | 1,000 | ,080 | -,117 | ,130 | ,464 | -,073 | ,074 |
| 5.It 5 T2 | ,105 | ,046 | -,014 | ,080 | 1,000 | -,096 | ,092 | ,066 | -,089 | ,189 |
| 6.It 6 T2 | -,096 | -,022 | ,045 | -,117 | -,096 | 1,000 | ,008 | -,015 | -,069 | -,020 |
| 7.It 7 T2 | ,092 | -,062 | ,073 | ,130 | ,092 | ,008 | 1,000 | ,151 | ,026 | ,357 |
| 8.It 8 T2 | ,110 | ,035 | -,146 | ,464 | ,066 | -,015 | ,151 | 1,000 | ,155 | ,045 |
| 9.It 9 T2 | -,008 | ,413 | -,105 | -,073 | -,089 | -,069 | ,026 | ,155 | 1,000 | -,060 |
| 10.It 10 T2 | ,042 | -,132 | ,015 | ,074 | ,189 | -,020 | ,357 | ,045 | -,060 | 1,000 |

Note: It 1 T2 = item 1 at time 2, etc
