# Supplementary material for: Cognitive synergy in groups and group-to-individual transfer of decision-making competencies
Source: Front Psychol. 2015 Sep 22;6:1375. doi: 10.3389/fpsyg.2015.01375 (PMC4585191; doi:10.3389/fpsyg.2015.01375)
Supplement: Supplementary file 2 [file DataSheet2.DOCX]

**Appendix**

**Decision-making tasks time 1**

1. Suppose you play a coin tossing game and flip a coin several times and for 9 times you are right calling “heads” (the coin falls on heads). What do you think will happen when you toss the coin for the 10^th^ time?

1. I will call “heads”.
2. I will call “tails”.
3. **Both sides are equally probable.**
4. I cannot decide.

2. Imagine that your country is preparing for the outbreak of an unusual Asian disease, which is expected to kill 600 people. Two alternative programs to combat the disease have been proposed: Program A and Program B. The exact scientific estimates of the consequences of the programs are known and your aim is to save as many people as possible. Choose one of the following alternatives?

1. If Program A is adopted, 200 people will be saved.
2. If Program B is adopted, there is a ⅓ probability that 600 people will be saved, and ⅔ probability that no people will be saved.
3. **Both programs are equally effective.**
4. I cannot decide.

3. A family has 10 kids and 9 of them are boys. The wife is pregnant again. Which do you think will most probably be the sex of the newborn child?

1. The newborn will be a boy.
2. The newborn will be a girl.
3. **Both are equally probable.**
4. I cannot decide.

4. Suppose you have an urn with 90 balls, 30 yellow and 60 red or blue. You can draw one ball from the urn and you have to bet on the color of the ball. If you are correctly guessing the color of the ball, you can earn 100$. Which color do you think has the highest probability of being drawn?

1. Yellow.
2. Red.
3. **Both have equal probability of being drawn.**
4. I cannot decide.

5. You have the chance of buying a lottery ticket. Suppose that on the first ticket the numbers are 7, 12, 18, 24, 33 and 45 and on the second ticket, the numbers listed are 1, 2, 3, 4, 5 and 6. Which one do you think has the highest chance of being winner?

1. The first ticket.
2. The second ticket.
3. **Both tickets have equal chances of being a winner.**
4. I cannot decide.

6. Linda is 31 years old, single, outspoken and very bright. She majored in philosophy. As a student, she was deeply concerned with issues of discrimination and social justice, and also participated in anti-war demonstrations. Which statement do you think has the highest chance of being correct?

1. **Linda is a bank teller.**
2. Linda is a feminist bank teller.
3. I cannot decide.

7. John lives in a village with many farmers and one librarian. John carries few books with him and wears glasses. Which statement do you think has the highest chance of being correct?

1. **John is a farmer.**
2. John is the librarian.
3. I cannot decide.

8. You take part in a contest in which you have to draw without seeing a ball from an urn. If you draw a red ball you will earn 100$. You can choose between 2 urns. Assuming that you would like to maximize your chances of drawing a red ball, what do you think which of the following alternatives will you select?

1. I would select the first urn, which contains 50 red and 50 black balls.
2. I would select the second urn, which contains 100 mixed red and black balls in an unknown proportion.
3. **In both urns I have equal probability of choosing a red ball.**
4. I cannot decide.

9. A leading commander and his army are in an ambush, which is expected to kill all 10.000 soldiers. The commander and his strategy advisors identified 2 possible ways out of the ambush: route X and route Y. The exact scientific estimates of the consequences of taking the two routes are known and your aim is to have as few casualties as possible. Choose one of the following alternatives?

1. If the army retreats on the route X, 7500 soldiers will die.
2. If the army retreats on the route Y, there is a ¼ probability that nobody will die and ¾ probability that all soldiers will die.
3. **Both routes are equally dangerous.**
4. I cannot decide.

10. Which event do you think is most probable?

1. **A person had a heart attack.**
2. A cigarette smoker over 50 had a heart attack.
3. I cannot decide.

**Decision-making tasks time 2**

1. Supposed that an unbiased coin is flipped three times and each time the coin lands on heads. If you had to guess the next toss, what do you think will happen?

1. I will call “heads”.
2. I will call “tails”.
3. **Both sides are equally probable.**
4. I cannot decide.

2. Imagine that recent evidence has shown that pesticide is threatening the lives of 1200 endangered animals. Two response options have been suggested: response A and response B. The exact scientific estimates of the consequences of the two response options are known and your aim is to save as many endangered animals as possible. Choose one of the following alternatives?

1. If Program A is adopted, 900 animals will be saved.
2. If Program B is adopted, there is a 75% chance that 1200 animals will be saved, and 25% chance that no animal will be saved.
3. **Both response options are equally effective.**
4. I cannot decide.

3. The mean IQ (intelligence quotient – intelligence level) of eight graders is known to be 100. You have selected a random sample of 50 children for a study of educational achievements. The first child to be tested has an IQ of 150. What do you expect to be the IQ for the whole sample?

1. 100.
2. 150.
3. **101.**
4. I cannot decide.

4. You are in a room with two large urns. The urns are covered and you cannot see inside them. You know that the urn on the left contains 50 white marbles and 50 black marbles. The urn on the right contains 100 marbles white and black randomly mixed. In which urn do you think you have the highest probability of picking a white marble?

1. Right urn.
2. Left urn.
3. **In both urns white marbles have equal probability of being drawn.**
4. I cannot decide.

5. You have the chance of playing a dice rolling game. Suppose that the two options (number sequences) on which you can bet are 5, 1, 4, 2, 3, 6 and 1, 2, 3, 4, 5, 6. Which one do you think has the highest chance of being winner?

1. The first number sequence.
2. The second number sequence.
3. **Both sequences have equal chances of occurring.**
4. I cannot decide.

6. Jim is tall, muscular and very athletic. He is also very competitive, drives an expensive car and he wears rather flashy clothing. Which statement do you think has the highest chance of being correct?

1. Jim is a professional athlete.
2. **Jim is a lawyer or financial analyst.**
3. I cannot decide.

7. Jack is a 45-year-old man. He is married and has four children. He is generally conservative, careful, and ambitious. He also shows strong interest in political and social issues. Which statement do you think has the highest chance of being correct?

1. **Jack is an engineer.**
2. Jack is an engineer and a political activist.
3. I cannot decide.

8. You take part in a contest in which you have to draw without seeing a ball from an urn. If you draw a red ball you will earn 100$. You can choose between 2 urns. Assuming that you would like to maximize your chances of drawing a red ball, what do you think which of the following alternatives will you select?

1. I would select the first urn, which contains 10 red and 10 black balls.
2. I would select the second urn, which contains 20 mixed red and black balls in an unknown proportion.
3. **In both urns I have equal probability of choosing a red ball.**
4. I cannot decide.

9. Imagine that a hospital is treating 32 injured soldiers, who are all expected to lose one leg. There are two doctors that can do the surgery: doctor A and doctor B. Their exact rates of surgery failure in similar cases are known and your aim is to save as many soldiers as possible. Choose one of the following alternatives?

1. If doctor A is hired, 20 soldiers will lose one leg.
2. If doctor B is hired there is a 62.5% chance that all soldiers will lose one leg and 37.5% chance that nobody will lose one leg.
3. **Both doctors are equally effective.**
4. I cannot decide.

10. Which event do you think is most probable?

1. **A person had a fatal car accident.**
2. A race driver had a fatal car accident.
3. I cannot decide.

**Notes**

Minor phrasing adjustments were made based on the suggestions received from reviewers, bolded alternatives are scored with 1, the remaining alternatives with 0. The bolded answers were deemed correct under the following assumptions: expected value is considered the normative standard, for conjunction fallacy we use a joint (rather than conditional) interpretation of probability and in the case of Ellsberg paradox we assumed an equal distribution of possibilities.
